# Supplementary material for: Embryo mechanics cartography: inference of 3D force atlases from fluorescence microscopy
Source: Nat Methods. 2023 Dec 6;20(12):1989–99. doi: 10.1038/s41592-023-02084-7 (PMC10703677; doi:10.1038/s41592-023-02084-7)
Supplement: Supplementary file 2 — Reporting Summary [file 41592_2023_2084_MOESM2_ESM.pdf]

nature portfolio

Corresponding author(s): Hervé Turlier

Last updated by author(s): Oct 2, 2023

Reporting Summary

Nature Portfolio wishes to improve the reproducibility of the work that we publish. This form provides structure for consistency and transparency in reporting. For further information on Nature Portfolio policies, see our Editorial Policies and the Editorial Policy Checklist.

Statistics

For all statistical analyses, confirm that the following items are present in the figure legend, table legend, main text, or Methods section.

n/a	Confirmed	
☐	☒	The exact sample size (*n*) for each experimental group/condition, given as a discrete number and unit of measurement
☒	☐	A statement on whether measurements were taken from distinct samples or whether the same sample was measured repeatedly
☒	☐	The statistical test(s) used AND whether they are one- or two-sided<br>*Only common tests should be described solely by name; describe more complex techniques in the Methods section.*
☒	☐	A description of all covariates tested
☒	☐	A description of any assumptions or corrections, such as tests of normality and adjustment for multiple comparisons
☐	☒	A full description of the statistical parameters including central tendency (e.g. means) or other basic estimates (e.g. regression coefficient) AND variation (e.g. standard deviation) or associated estimates of uncertainty (e.g. confidence intervals)
☒	☐	For null hypothesis testing, the test statistic (e.g. *F*, *t*, *r*) with confidence intervals, effect sizes, degrees of freedom and *P* value noted<br>*Give P values as exact values whenever suitable.*
☒	☐	For Bayesian analysis, information on the choice of priors and Markov chain Monte Carlo settings
☒	☐	For hierarchical and complex designs, identification of the appropriate level for tests and full reporting of outcomes
☒	☐	Estimates of effect sizes (e.g. Cohen's *d*, Pearson's *r*), indicating how they were calculated

Our web collection on statistics for biologists contains articles on many of the points above.

Software and code

Policy information about availability of computer code

Data collection	No specific code was used to collect data.
Data analysis	Python >3.6 and classical associated scientific libraries (NumPy >1.25, SciPy >1.11, NetworkX >3.1, Matplotlib, scikit-image >1.0) were used for data analysis. Polyscope >1.3.0 (https://polyscope.run) was used to build a custom viewer for visualizing inference results on reconstructed meshes. CGAL 5.5.1 (https://www.cgal.org) was used to compare our mesh reconstruction with state-of-the-art techniques from computer graphics.<br>We developed two custom codes, that will be available on our GitHub repository and on the Python package index PyPi (https://pypi.org) upon publication:<br>- the Delaunay-watershed code for mesh reconstruction from segmentation masks: https://github.com/VirtualEmbryo/delaunay-watershed<br>- the foambryo code for 3D force inference and visualization: https://github.com/VirtualEmbryo/foambryo

For manuscripts utilizing custom algorithms or software that are central to the research but not yet described in published literature, software must be made available to editors and reviewers. We strongly encourage code deposition in a community repository (e.g. GitHub). See the Nature Portfolio guidelines for submitting code & software for further information.

Data

Policy information about availability of data

All manuscripts must include a data availability statement. This statement should provide the following information, where applicable:

- Accession codes, unique identifiers, or web links for publicly available datasets
- A description of any restrictions on data availability
- For clinical datasets or third party data, please ensure that the statement adheres to our policy

Images and segmentation masks are already available publicly for P. Mammillata embryos on figshare (≥ 64 cells) (https://figshare.com/projects/Phallusia_mammillata_embryonic_development/64301) and for C. elegans embryos on figshare (https://figshare.com/articles/journal_contribution/CShaper_Supplementary_Data/12839315). The simulated dataset (artificial images, segmentation masks and tensions/pressures) used to benchmark the method is available publicly on Zenodo (https://zenodo.org/record/7881017). Additional experimental images of ascidian embryos ($<$ 64 cells) and their segmentation masks are available upon request.

Human research participants

Policy information about studies involving human research participants and Sex and Gender in Research.

Reporting on sex and gender	N/A
Population characteristics	N/A
Recruitment	N/A
Ethics oversight	N/A

Note that full information on the approval of the study protocol must also be provided in the manuscript.

Field-specific reporting

Please select the one below that is the best fit for your research. If you are not sure, read the appropriate sections before making your selection.

☒ Life sciences ☐ Behavioural & social sciences ☐ Ecological, evolutionary & environmental sciences

For a reference copy of the document with all sections, see nature.com/documents/nr-reporting-summary-flat.pdf

Life sciences study design

All studies must disclose on these points even when the disclosure is negative.

Sample size	Figures 4, 5 and 6b: among n>3 samples we provided the most representative microscopy image or mechanical pattern. No averaging of mechanical maps was performed; the comparisons of myosin and surface tension patterns (Figure 5c) are shown for illustrative purpose.
Data exclusions	We did not exclude data: for instance in Extended data Figure 5a, we present most of the embryos available in the public repository. For other figures, we selected representative mechanical maps inferred from microscopy images based on their relevance to published data or emerging patterns.
Replication	We found quantiatively consistent replicates for the mechanical patterns observed in Figures 4e (n>3), Figure 5 and Figure 6b
Randomization	We did not allocate samples to different groups, but retained all imaged embryos displaying a normal development.
Blinding	N/A

Reporting for specific materials, systems and methods

We require information from authors about some types of materials, experimental systems and methods used in many studies. Here, indicate whether each material, system or method listed is relevant to your study. If you are not sure if a list item applies to your research, read the appropriate section before selecting a response.

Materials & experimental systems

n/a	Involved in the study
☒	☐ Antibodies
☒	☐ Eukaryotic cell lines
☒	☐ Palaeontology and archaeology
☐	☒ Animals and other organisms
☒	☐ Clinical data
☒	☐ Dual use research of concern

Methods

n/a	Involved in the study
☒	☐ ChIP-seq
☒	☐ Flow cytometry
☒	☐ MRI-based neuroimaging

Animals and other research organisms

Policy information about studies involving animals; ARRIVE guidelines recommended for reporting animal research, and Sex and Gender in Research

Laboratory animals	The study did not involve laboratory animals
Wild animals	Adults from the ascidian specie Phallusia mammillata were collected in Sète by scuba divers and transported to the laboratory in Villefranche-sur-mer in a tank of seawater at 16°C by car (371km). Animals are dissected to collect their eggs and sperm.
Reporting on sex	N/A
Field-collected samples	The eggs of the ascidian Phallusia mammillata were harvested from animals and kept in the laboratory in a tank of natural seawater at 16°C.
Ethics oversight	No ethical approval or guidance required. All protocols are published: https://doi.org/10.1007/978-1-61779-210-6_14

Note that full information on the approval of the study protocol must also be provided in the manuscript.
